# DEMENTIA-FOCUSED PERSON-DIRECTED CARE TRAINING IN THE NURSING HOME: FIDELITY AND OUTCOMES

**DOI:** 10.1093/geroni/igz038.1394

**Published:** 2019-11-08

**Authors:** Joann P Reinhardt, Orah Burack, Verena Cimarolli, Audrey Weiner

**Affiliations:** 1 The New Jewish Home, New York, New York, United States; 2 Leading Age, Washington, District of Columbia, United States

## Abstract

To address the behavioral health of nursing home (NH) residents living with dementia, training direct care staff (DCS) is essential, for the well-being of both residents and staff. We evaluated a training for DCS focused on providing care for persons with advanced dementia who are at-risk of not having care needs met, largely due to communication deficits. Staff were trained in promoting comfort and reducing distress through person-directed care (PDC), deeply knowing each resident, and anticipating needs. Subsequent fidelity interviews with staff showed a higher number of PDC practices utilized by staff in the intervention communities compared to usual care. We also compared the impact of the PDC model versus a traditional model of NH care on resident clinical outcomes, finding a significant interaction where those in the intervention group had fewer clinical symptoms over a 6-month period. Implications for training in the NH setting will be discussed.

